# Web of Objects Based Ambient Assisted Living Framework for Emergency Psychiatric State Prediction

**DOI:** 10.3390/s16091431

**Published:** 2016-09-06

**Authors:** Md Golam Rabiul Alam, Sarder Fakhrul Abedin, Moshaddique Al Ameen, Choong Seon Hong

**Affiliations:** Computer Science and Engineering, Kyung Hee University, 1732 Deokyoungdaero, Gilheung-gu, Yongin-si 446-701, Korea; robi@khu.ac.kr (M.G.R.A.); saab0015@khu.ac.kr (S.F.A.); ameen@khu.ac.kr (M.A.A.)

**Keywords:** ambient assisted living, web of objects, mental healthcare, emergency psychiatry, smart home

## Abstract

Ambient assisted living can facilitate optimum health and wellness by aiding physical, mental and social well-being. In this paper, patients’ psychiatric symptoms are collected through lightweight biosensors and web-based psychiatric screening scales in a smart home environment and then analyzed through machine learning algorithms to provide ambient intelligence in a psychiatric emergency. The psychiatric states are modeled through a Hidden Markov Model (HMM), and the model parameters are estimated using a Viterbi path counting and scalable Stochastic Variational Inference (SVI)-based training algorithm. The most likely psychiatric state sequence of the corresponding observation sequence is determined, and an emergency psychiatric state is predicted through the proposed algorithm. Moreover, to enable personalized psychiatric emergency care, a service a web of objects-based framework is proposed for a smart-home environment. In this framework, the biosensor observations and the psychiatric rating scales are objectified and virtualized in the web space. Then, the web of objects of sensor observations and psychiatric rating scores are used to assess the dweller’s mental health status and to predict an emergency psychiatric state. The proposed psychiatric state prediction algorithm reported 83.03 percent prediction accuracy in an empirical performance study.

## 1. Introduction

Incorporating smartness in the home environment ensures security, comfort and healthcare, which are the primary goals of Ambient Assisted Living (AAL) [1]. The proliferation of wearable sensor technology has elevated the AAL systems for personalized care at home and in mobile environments. A number of activity recognition [2] and fall detection [3] methods have been proposed for monitoring physical health in the smart home [4] environment. Yet, mental [5] and cognitive health [6] status are not explored to such an extent for assisted living. Therefore, this paper proposes an emergency psychiatric state prediction method for the AAL environment.

Moreover, the traditional Service-Oriented Architecture (SOA) [7], which ignores the layered design approach, is proven to be too heavyweight for the resource constraints of a smart home environment to enable AAL service. Therefore, this paper proposes an AAL framework based on a Web of Objects (WoO) [8,9], especially for the mental healthcare scenario. Dwellers’ or patients’ major psychiatric symptoms are monitored through home-based lightweight biosensors and web-based psychiatric rating scales (e.g., the Beck Depression Inventory (BDI) [10], the Hamilton Rating Scale for Depression (HRSD) [11]). The biosensor observations and psychiatric rating scores are used to assess the dweller’s mental health status and thus to predict an emergency psychiatric state.

The emergency psychiatric states of patients’ are modeled as a discrete set of states in a Hidden Markov Model (HMM) [12]; objectified sensor observations and patients’ history are considered to be observations for the HMM. Then, based on those observations, Viterbi [12], a machine learning algorithm, is used to generate the most probable psychiatric state sequence. The prognosis of an emergency psychiatric state is determined from the most likely psychiatric state sequence, using the proposed psychiatric state prediction algorithm.

The major contributions of this research are (1) the ambient assisted living framework and (2) emergency psychiatric state prediction:

1. Ambient assisted living framework: A web of objects-based smart home framework is presented in Section 3 for in-home personalized psychiatric care. In order to predict and monitor an emergency psychiatric state, collaboration among the objects is required. Therefore, a mental healthcare ontology is developed for presenting and extracting semantic relationships among the web of objects in a psychiatric care scenario. The framework enables a platform to cooperate, harmonize and share the mental healthcare objects for ambient assisted living services (e.g., emergency psychiatry).
2. Emergency psychiatric state prediction: Decoding the psychiatric state of a patient through indirect channels of non-invasive biosensors is challenging. The discriminative features of putative risk factors for emergency psychiatric states are extracted from biosensor observations. For structured prediction, the emergency psychiatric states are modeled through an HMM. The psychophysiological features along with the psychometric features and patient histories are used as the observations to predict psychiatric state. Additionally, an emergency psychiatric state prediction algorithm is proposed for inferring the risk of psychiatric emergency. The probabilistic model parameters are estimated, and the accuracy of the model is validated over a training and testing dataset. The prototype is developed and tested.

The remaining sections of this paper are organized as follows: Section 2 reviews the related works; Section 3 discusses the details of the web of objects-influenced AAL framework and the emergency psychiatric state prediction model. Section 4 shows the prototype implementation and performance evaluation. Section 5 concludes the paper with some future directions.

## 2. Literature Review and Related Works

This section discusses the concept of emergency psychiatry and the state-of-the-art technologies in mental healthcare and ambient assisted living.

### 2.1. Emergency Psychiatry

**Figure 1:**
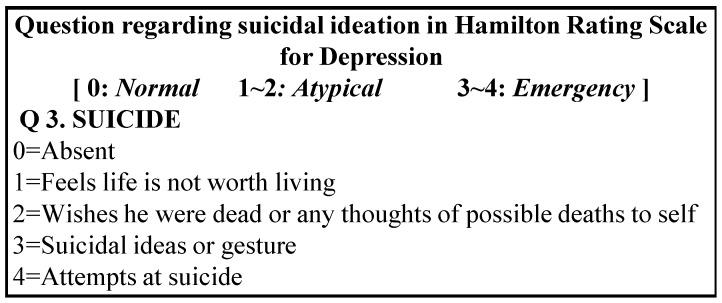
Question Number 3 of the Hamilton Rating Scale for Depression.

Emergency psychiatry deals with psychiatric emergencies, which are acute disturbances of behavior, thought and mood in mentally-disordered patients, who are at risk of potential danger to themselves (e.g., suicide) and to others (e.g., homicide) [13]. However, this research is focused only on the suicidal psychiatric emergency due to the unavailability and unobtainability of datasets of the homicide psychiatric emergency. The higher severity levels of stress, depression, hopelessness, aggressiveness and anxiety are the most influential features of a psychiatric emergency as presented in the clinical model of suicidal behavior [14]. Thus, different psychiatric scales, such as the Beck Depression Inventory (BDI) [10] and the Beck Hopelessness Scale (BHS) [15], are used to measure the severity levels of potential triggers for an emergency psychiatric state. However, those scales are insufficient for real-time and continuous assessment of the putative risk factors of an emergency psychiatric state. Therefore, in this research, the non-invasive and wearable biosensor signals are analyzed by utilizing an AAL framework to predict the emergency psychiatric state. Therefore, the psychiatric states are defined objectively as Normal (N), Atypical (A) and Emergency (E), based on Question Number 3 of the Hamilton Rating Scale for Depression (HRSD) [11], as presented in Figure 1.

### 2.2. Related Works

Some of the legendary research and developments regarding mental healthcare [16,17] are studied in this section. The MONitoring, treAtment and pRediCtion of bipolAr (MONARCA) disorder episodes is a European project, which opens the door for self-management, assessment and treatment of mentally sick bipolar patients using some tiny sensors [18]. The wearable system consists of a smart phone, wrist-worn sensors for monitoring patient’s activity and smart socks to recognize the mental states of bipolar patients. The project is especially developed for monitoring the episodes of bipolar disorder patients, where they consider manic episode, mild depression episode and severe depression episode as the mental states of bipolar patients. Basically, the MONARCA is a pioneering research that uses wearable sensors (e.g., GPS and accelerometer) to monitor psychiatric patients. However, the MONARCA did not consider the psychophysiological observations through biosensors to predict the manic and depression states of bipolar disorder patients. On the other hand, a novel emergency psychiatric state (i.e., atypical, emergency and normal) prediction method for AAL is proposed in this paper, where the EDA, ECG, EMG and BVP sensor observations are used for determining the potential triggers of psychiatric emergencies.

Dobscha et al. [19] study the feasibility of an in-home telehealth system to monitor the depression and pain of psychiatric patients. The collected psychiatric rating scores for depression measurement (Patient Health questionnaire (PHQ-9)) [20] and pain severity measurement through the short form 36-items health survey questionnaire for veterans (SF36-V) [21] shows promising results in the case of the remote psychiatric care system. The study recommends home the health monitoring system to observe the symptom severity of the mental health condition for both clinical and research purposes.

Niculescu et al. [22] successfully classified the blood biomarkers to recognize suicidality in the patients with mood disorder. Therein, the authors used convergence functional genomics to classify the genes responsible for suicidality and identified spermidine/Spermine N1-Acetyltransferase 1 (SAT1) as the most influential blood biomarker among other blood biomarkers. The drawback of the blood biomarker-based suicidality prediction is its invasiveness, i.e., one needs the blood to investigate the suicidality biomarkers. However, the suicidal genome classification is only possible in an intensive clinical and pathological setup environment. Thus, it is infeasible to adopt the technique in an Ambient Intelligence (AmI) system for continuous monitoring of psychiatric patients or elderly people in the home environment.

There has been some influential research to measure the stress, depression, anxiety, and frustration levels of patients through biosensors. Cornella et al. [23] used Electro-Dermal Activity (EDA) sensors to observe the skin conductance response (i.e., sympathetic arousal) to determine the stress levels of patients. Imaoka et al. [24] used Electrocardiogram (ECG) sensors to measure the coefficient of variations of consecutive 100 R-Rintervals, which is an objective index of depression. Using ECG sensors, Reilly et al. [25] observed lengthening QTc-intervals (rate-corrected QT) in case of drug-induced arrhythmia. The Blood Volume Pulse (BVP) sensor is used to measure heart rate, heart rate variability, inter-beat interval and the deviation in BVP amplitude. Riseberg et al. [26] measured the frustration and anxiety levels of a patient using Galvanic Skin Response (GSR), BVP and Electromyography (EMG) sensors.

**Figure 2:**
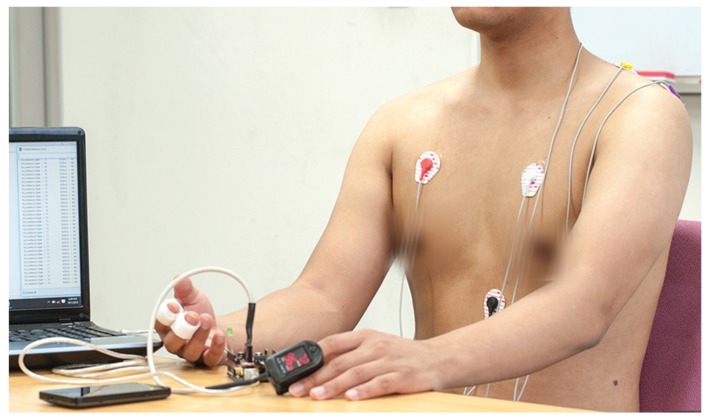
Bio-sensors network to collect psychophysiological observations in an AAL framework.

However, this paper deals with the emergency psychiatric state detection through feature level fusion of biosensors and psychometric observation and patient histories. In this proposal, four kinds of biosensors (i.e., Electro-Dermal Activity sensor (EDA), Electrocardiogram sensor (ECG), Blood Volume Pulse sensor (BVP) and surface Electromyography sensor (EMG) observation are used to infer the emergency psychiatric state, as shown in Figure 2.

Most of these bio sensors are lightweight and readily available with consumer products (e.g., smartphones, smart wrist bands, chest belt and bracelet). Recently, BVP sensors have been embedded in smart phones where the camera is used to pass optical rays for the measurement of pulse and oxygen saturation level in the blood. Some of the EDA sensors are embedded with smartwatches and wristbands as consumer products for measuring a subject’s stress level. The ECG and EMG sensors are also available as a chest belt, arm band and smart vest. Each of the aforementioned sensors have either their own communication capability or they use their host as the gateway node to communicate with the Internet. However, these sensors may also be included in health kits for assisted living as mass-marketed consumer products.

## 3. Materials and Methods

This section has two broad subsections. The proposed WoO-based AAL framework is discussed in Section 3.1. On top of the WoO-based AAL framework, the psychiatric state prediction method is developed, which is discussed in Section 3.2.

### 3.1. Web of Objects-Based Smart Home Framework for Ambient Assisted Living

**Figure 3:**
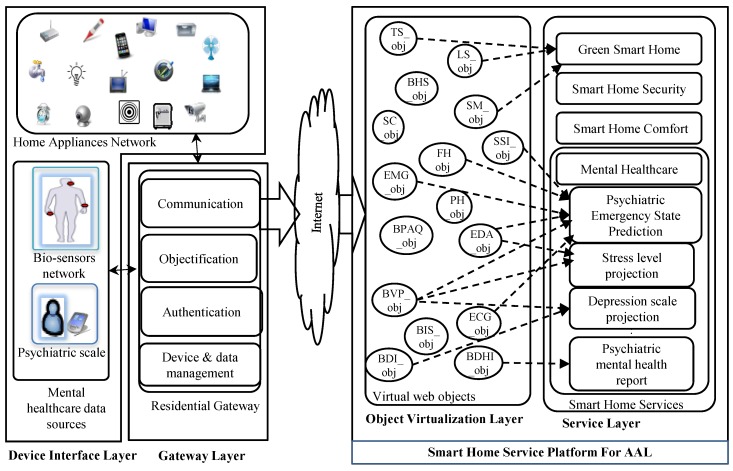
Web of Objects (WoO)-based smart home framework for ambient assisted living. Different home appliances and sensors are objectified as web of objects and virtualized in the virtualization layer. The virtualized web objects are defined as: TS, Temperature Sensor; LS, Light Sensor; BHS, Beck Hopelessness Scale [15]; FH, Family History; PH, Patient’s History; SM, Smart Meter; SC, Smart Camera; BPAQ, Buss–Perry Aggression Questionnaire [27]; BIS, Barratt Impulsiveness Scale [28]; BDHI: Buss–Durkee Hostility Inventory [29]; SSI, Scale of Suicide Ideation [30].

The traditional SOA [18], which ignores the layered design approach, is proven as too heavyweight for the resource constraints of a smart home environment to enable AAL service. Thus, the web service technology-based framework is proposed for facilitating a simple and open management platform for heterogeneous entities and for service creation and composition, where objects are abstracted as web services. The generic smart home framework is presented in Figure 3, and the description of each layer is explained in this section from the perspective of a mental healthcare scenario.

#### 3.1.1. Device Interface Layer

To enable AAL service, this layer consists of the physical sensors and actuators for an intelligent system. The home appliance network [9] includes a biosensor network with a sink node (e.g., mobile sink) [31], where body sensors are connected through wireless communication protocols to collect psychophysiological data. The smart phone- or smart TV-based web interface for psychiatric disorder screening scales (e.g., BDI [10]) is also embedded with the home appliance network to collect psychometric data. Therefore, the biosensor network and psychiatric assessment scales of the device interface layer of the AAL framework enable the collection of real-time and continuous observations from patients.

#### 3.1.2. Gateway Layer

**Figure 4:**
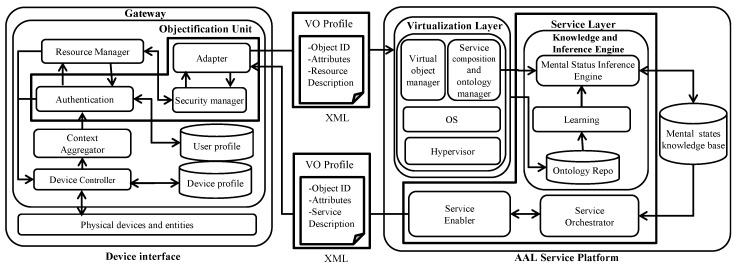
Functional modules of a web of object-based AAL platform especially in a mental healthcare scenario.

The gateway layer bridges the AAL service platform and device interfaces. The core objective of this module is to provide device management, user profile and device profile management along with web connectivity to all of the devices. In addition, the gateway aggregates the environmental data generated by the heterogeneous consumer devices and sensors, which are disseminated as a web of objects (i.e., XML) for processing afterwards in the virtualization layer, as presented in Figure 4.

#### 3.1.3. Object Virtualization Layer

**Figure 5:**
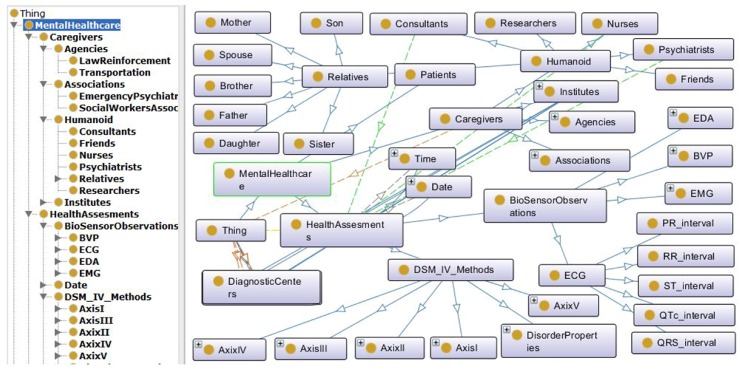
The mental healthcare semantic ontology for emergency psychiatry in an AAL platform. The service layer of the proposed web of object-based AAL framework uses this semantic ontology to create ambient assisted services, such as emergency psychiatric state prediction. According to psychiatric state model presented in Figure 6, besides sensor observations, the scores of the psychiatric rating scale, patient’s personal, medical and family histories are necessary for emergency psychiatric mental state prediction. The ontology presented in this figure shows the semantic relationship among those objects. The ontology is also used to multicast messages to patient’s relatives, friends and caregivers in the case of emergency.

The objectified physical devices and entities are virtualized in this layer to simplify the object collaboration for service creation. Consequently, this procedure also enables the devices to be identified and actuated through their own device profile, method and control information. In fact, all of the virtual objects maintain a semantic relationship with each other. The mental healthcare semantic ontology [32] is developed and presented in Figure 5 for emergency psychiatric service creation. The developed psychiatric ontology is based on the Diagnostic and Statistical Manual of Mental Disorders IV, Text Revision (DSM-IVTR) [33].

#### 3.1.4. Service Layer

The service layer is responsible for the on-demand and pro-active service creation. In the exemplary mental healthcare scenario, the service layer creates a pro-active, psychiatric emergency state prediction service to serve users and emergency care authorities. The knowledge and inference engine (see Figure 4) of this layer acts as the brain of the proposed AAL framework to enable personalized mental healthcare service. The service layer can also provide on-demand services; for example, by sending a service request using a mobile application or mobile web browser, users can get the mental health report on stress and depression. The semantic ontology model in the object virtualization layer is used for psychometric and psychophysiological feature extraction. An example query of the psychometric feature extraction is given as follows:

**Query:** Find the family history of the patient ‘G.H.Park’, to know whether or not she has a family history of ‘Suicide’.

*PREFIX men:<http://semanticsweb.org/ontologies/mentalhealth.owl>*
*PREFIX kosis:<http://kosis.kr/DeathsByCause/rdf-schema> Select? FamilyHistory Where? FamilyHistory*
*men:G.H.Park men: Relatives koisis:DeathCause*
		  

**The SPARQL query results:**
|———————-|
| Family History |
|———————-|———————————————————|
|men:Father: C.H. Park koisis:DeathCause:Assassination |
|men:Mother: Y.S. Yuk koisis:DeathCause:Assassination |
|men:Brother: J.M. Park koisis: DeathCause: |
|men:Sister: G.Y. Park koisis: DeathCause: |
|———————————————————————————|

The developed mental healthcare ontology of the WoO-based AAL framework facilitates such queries for extracting the feature values needed to predict the emergency psychiatric sate of patients.

### 3.2. Prediction of Psychiatric State

The proposed AAL framework provides a platform for assessing and monitoring the mental health of inhabitants in a smart home environment. It also infers emergency psychiatric states to complement emergency psychiatric care service. Therefore, this subsection discusses the details of proposed psychiatric state prediction methods.

#### 3.2.1. Emergency Psychiatric State Modeling

The psychiatric states are considered as hidden or latent variables to model the emergency psychiatric states, because these states are not fully or partially observable. The hidden states can be predicted through some biosensor observations, psychiatric screening scores, individual psychiatric records and family histories. The future psychiatric mental state of a patient is dependent exclusively on the current state. Thus, the HMM [34,35] is barely apposite to model the psychiatric states of individuals.

**Figure 6:**
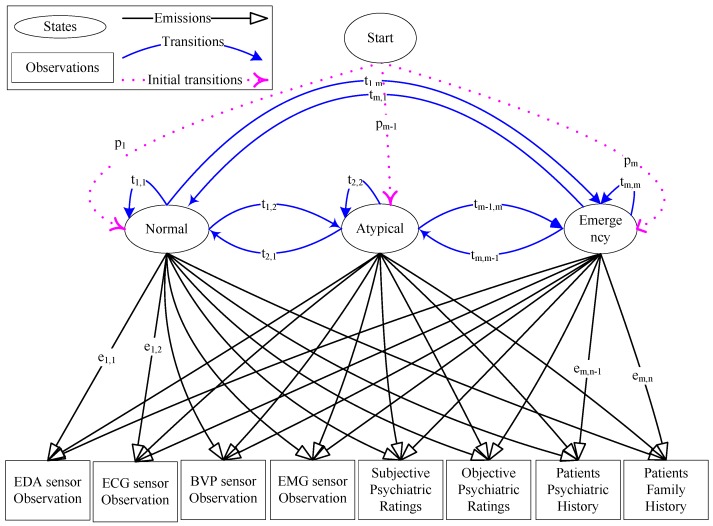
Hidden Markov model-based psychiatric state model for predicting an emergency psychiatric state in the AAL framework.

A total of *m* states is considered in a discrete time Markov process, and a set of states is defined as S= {$s_{1}$, $s_{2}$, …, $s_{m}$}, where all states are hidden. The observed feature values of four biosensors, the scores of psychiatric assessment scales and historical observations of patients’ psychiatric state, personal and family histories are considered as the HMM observation set, as shown in Figure 6. The symbolic representation of the observations at time *t* is $O_{t}=$ {$o_{1}$, $o_{2}$, …, $o_{n}$}, where *n* is the number of total observations in which $o_{i}$ contains the observed feature values.

Now, the primary goal is to determine the most probable psychiatric state sequence Q= {$q_{1}$, $q_{2}$, …, $q_{p}$} ∈S based on the perceived observation sequence V= {$O_{t}$, $O_{t+\tau }$, $O_{t+2\tau }$, …, $O_{T}$} for a given time duration T= *t* + (w−1)*τ*, where *τ* is the time duration of each individual observation and *w* is the size of the observation window.

The defined HMM has three tuples, λ= {*π*, $T^{\prime }$, $E^{\prime }$}, where π= {$\rho_{1}$, …, $\rho_{m}$} is the set of initial state probabilities, $T^{\prime }=$ {$t_{1,1}$, $t_{1,2}$, …, $t_{m,n}$} are the transition probabilities and $E^{\prime }=$ {$e_{1,1}$, $e_{1,2}$, …, $e_{m,n}$} are the emission probabilities.

#### 3.2.2. Data Collection

A dataset [36] of 55 subjects was collected through a WoO-based AAL platform to prepare a mentally-disordered patient dataset for studying emergency psychiatric states. The subjects were recruited through mental health caregiver’s referral. Among the subjects, there were a total number of 36 males and 19 females of ages between 20 and 66 years. The research goals were explained to the individual subjects, and a signed consent, which included the authorization of data usage for research purposes, was obtained. An expert psychiatrist guided the data collection session. At first, the subjective ratings of physical life events (e.g., BDI) and traits (e.g., BIH) were collected through the mobile web-based benchmarked questionnaires [10,14,15,30]. Meanwhile, the objective ratings (e.g., HRSD) [11] of life events were also collected in parallel from first-degree relatives. The non-invasive biosensors were placed on the subject’s body to collect psychophysiological observations. Subsequently, consultation sessions between 45 to 135 min were arranged with the psychiatrist to detect the psychiatric state of patients. During these consultation sessions, the psychiatrist made remarks on the patient’s status at every 15-min interval. Initially, the psychiatrist categorized 14 patients as emergency, 16 patients as atypical and 25 as normal state, especially based on the HRSD, as shown in Figure 1.

#### 3.2.3. Feature Extraction

**Figure 7:**
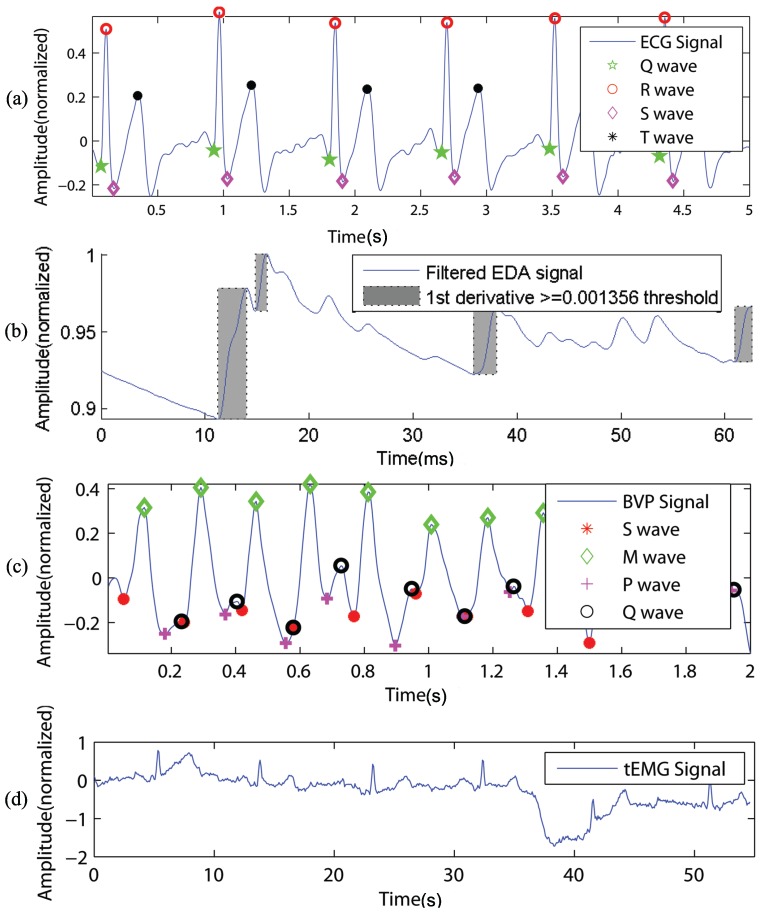
Preprocessed and extracted feature of (**a**) ECG; (**b**) EDA; (**c**) BVP and (**d**) EMG sensors.

The received signals from biosensors through the WoO interface were filtered using band pass filtering, so as to eradicate baseline wanders and high frequency noises. A window of 15 samples was considered for smoothing in determining the moving averages. Subsequently, the sensible features, such as the psychophysiological markers for risk factors of an emergency psychiatric state, were extracted from the preprocessed and denoised bio-signals, as shown in Figure 7. The R-R intervals, QRSamplitude and duration, QT interval, QTc interval, R/Sratio and R-peak were studied from the ECG signals, and the mean, variance, first and second derivatives [34] with respect to the time of those patterns were considered as the functional features [37,38,39].

The tonic level and phasic change corresponding to the electro-dermal components were studied from EDA signals. The mean values of Skin Conductance Level (SCL) and amplitude of Skin Conductance Responses (SCR) with time duration and frequency were considered as the functional features of EDA [37,40]. The blood volume and blood flow through the capillary bed of the skin were measured by the BVP sensor using a photoplethysmography mechanism. The signal waves of the BVP sensor are shown in Figure 7c, where S is the systolic valley, M is the systolic peak, P is the dicrotic notch and Q is the diastolic peak [41]. The measured values of the M-M interval, pulse width and area, M-Q interval, pulse interval, systolic amplitude, augmentation index, stiffness index and diastolic amplitude were used along with their means and standard deviations as the features from BVP signals [41,42,43].

The electrical activity of trapezius muscle was measured through surface EMG, which comprises the prevailing features of mental stress and frustration. The mean amplitude, mean and median frequency, average EMG gaps and the percentile of EMG gaps were extracted as the potential features from the EMG signal [44]. In order to form a complete feature vector, the biosensor feature vector was integrated with the patient’s medical, personal and family records and the psychiatric scales’ rating scores. The feature values of the feature vector were considered as observations throughout the paper.

**Figure 8:**
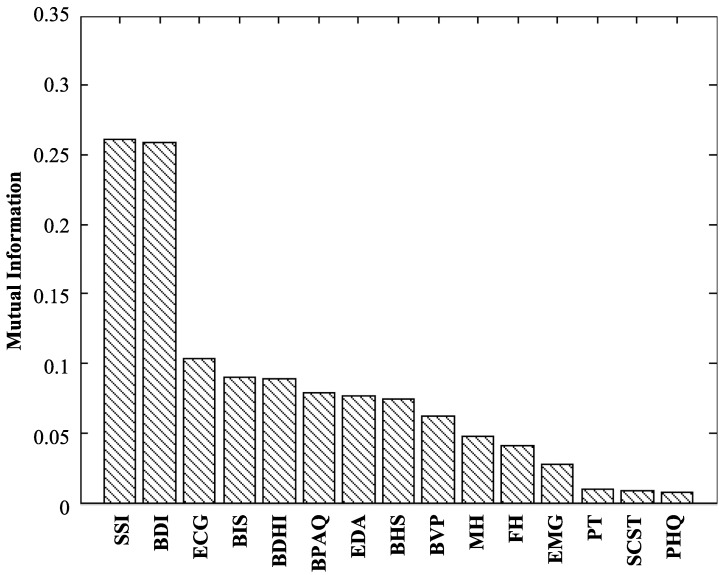
Discriminating feature groups based on mRMR. The cumulative mutual information of different features of Scale of Suicide Ideation (SSI) [30], Beck Depression Inventory (BDI) [10], Electrocardiogram (ECG), Baratt’s Impulsiveness Scale (BIS) [28], Buss-Durkee Hostility Inventory (BDHI) [29], Buss-Perry Aggression Questionnaire (BPAQ) [27], Electro-Dermal Activity (EDA), Beck Hopelessness Scale (BHS) [15], Blood Volume Pulse (BVP), patients’ Medical History (MH), patients’ Family History (FH), Electromyography (EMG), Personal Traits (PT), Stress and Coping Self-Test (SCST) [45] and Patient Health Questionnaire (PHQ) [20] are presented for the feature selection.

The observed psychiatric rating scores were quantified based on the benchmark psychiatric scales. Furthermore, the feature values are normalized through the min-max normalization [46] method. However, the minimal-Redundancy-Maximal-Relevance (mRMR) [47] feature selection method was applied to select a compact discriminating feature set based on the statistical dependency of features on emergency, atypical and normal psychiatric states. Figure 8 shows the 15 superior feature groups with higher mutual information based on mRMR, where it shows SSI, BDI and ECG features as the higher discriminating feature groups. Therefore, the features with a mutual information value of zero are discarded as non-discriminating futures to build up the compact candidate feature set.

**Figure 9:**
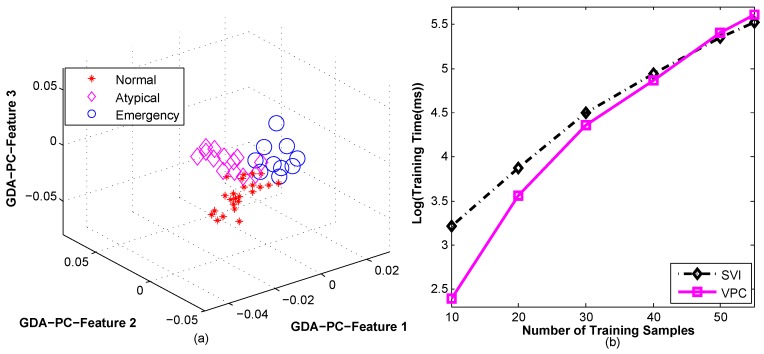
(**a**) Plotting of GDA-PC features of three types of psychiatric state class using HMM as a classifier; and (**b**) comparison of training times between VPC- and SVI-based HMM training.

Subsequently, Principal Component Analysis (PCA) [48] was performed for further dimension reduction in the transformed domain. Therefore, the individual contribution of signal features for psychiatric state prediction become indistinctive because of the transformed feature domain. In fact, PCA is a prominent subspace projection method that is widely used for reducing the dimension space from a higher dimension, as well as for maintaining the high order relationship. Afterwards, the Generalized Discriminant Analysis (GDA) [49] was applied on the Principal Component (PC) feature vectors for concentrating more closely the features of the same psychiatric state classes. The PCA and GDA dimension reduction methods are discussed elaborately in the previous studies [48,49]. However, Figure 9a shows the classification of psychiatric states based on the first three GDA PC features, where HMM is used as a classifier.

#### 3.2.4. Training and Validation

Once the dataset [36] with compact effective discriminating GDA-features was ready, the next step is to train the system. As the emergency psychiatric states are modeled through HMM, the goal of training is to learn the HMM parameters. The Viterbi Path Counting (VPC) algorithm [12] is used to determine the initial state probabilities, along with the transition and emission probabilities. The VPC training algorithm considers the whole dataset in a single pass to learn the HMM parameters. This makes the VPC not scale well to a large dataset. Additionally, for the sake of the scalability of real-world applications with millions of users, the Stochastic Variational Inference (SVI)-based HMM training algorithm [50] is used to determine the HMM parameters. The SVI-based HMM subsamples the training data and estimates the noisy HMM parameters for the subset. The iterative subsampling and parameter upgradation policy of SVI converges the noisy estimation of HMM parameters toward near optimal. The SVI-based HMM algorithm is presented and discussed in [50]. Figure 9b shows the lower training times of SVI compared to VPC with the increasing number of sample sizes. In the model construction, the 5-fold cross-validation is applied in the training phase for minimizing the over-fitting problem.

#### 3.2.5. Prediction of Emergency Psychiatric State

The trained system was therefore ready for real-time prediction of a patient’s psychiatric state. The biosensor observations, psychometric observations and patient histories were collected through the proposed assisted living framework presented in Section 3.1. The discriminating features were extracted from the collected observations according to the feature extraction principle presented in Section 3.2.3. The biosensor observations of *T* duration were necessary to make an effective prediction of a patient’s psychiatric state. The total *T* duration was subdivided into *τ* equivalent time intervals. Therefore, it was considered that the observations $O_{t}$, $O_{t+\tau }$, $O_{t+2\tau }$, …, $O_{T}$ were taken from patients at time slots *t*, t+τ, t+2τ, t+3τ, …, *T*; this can be rewritten as $t_{1}$, $t_{2}$, …, $t_{T}$ for simplicity. Hence, the sequence of perceived observations V= {$O_{t_{1}}$, $O_{t_{2}}$, …, $O_{T}$} in the respective time slots {$t_{1}$, $t_{2}$, …, $t_{T}$} made the problem suitable for applying the Viterbi algorithm to find out the most likely state sequence using trained HMM parameters *λ*.

**Figure 10:**
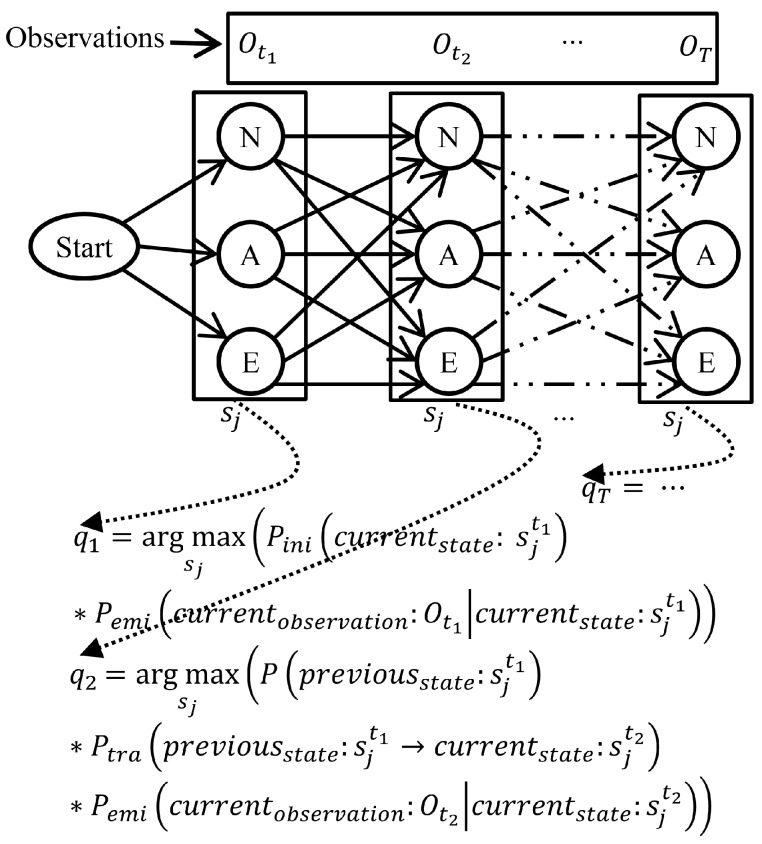
Trails of prediction of the psychiatric state from the observation of bio-signals and supporting features through the Viterbi algorithm [12].

The feature values (or observations) of bio-signals along with the supporting features of the complete feature vector contributed to predicting the psychiatric state of different time slots. The initial ($P_{ini}$), transition ($P_{tra}$) and emission ($P_{emi}$) probabilities of trained HMM were used in predicting the psychiatric state of time slot $t_{i}$ for observation $O_{t_{i}}$. Figure 10 shows the trails of predicting state sequence Q= {$q_{1}$, $q_{2}$, …, $q_{p}$} from the observation sequence V= {$O_{t_{1}}$, $O_{t_{2}}$, …, $O_{T}$} of different time slots through (1), where normal, atypical and emergency states were represented as *N*, *A* and *E*, respectively. Therefore, the Viterbi algorithm determined the most likely state sequence Q= {$q_{1}$, $q_{2}$, …, $q_{p}$} ∈S based on the perceived observation sequence V= {$O_{t_{1}}$, $O_{t_{2}}$, …, $O_{T}$} as presented in Step 1 of Algorithm 1 and in Row 1 of Table 1.

(1) $$q_{i}=\underset{s_{j}\in S}{\mathrm{argmax}}\left\{\begin{array}{c} P_{ini}\left(s_{j}^{t_{i}}\right)*P_{emi}\left(o^{t_{i}}|s_{j}^{t_{i}}\right);\;\;\;If\left(i=1\right)\\ P\left(s_{j}^{t_{i-1}}\right)*P_{tra}\left(s_{j}^{t_{i-1}},s_{j}^{t_{i}}\right)*P_{emi}\left(o^{t_{i}}|s_{j}^{t_{i}}\right);\;\;\;If\left(i>1\right)\\ 0,\;\;\;otherwise \end{array}\right.$$

**Algorithm 1:** Psychiatric state prediction. 
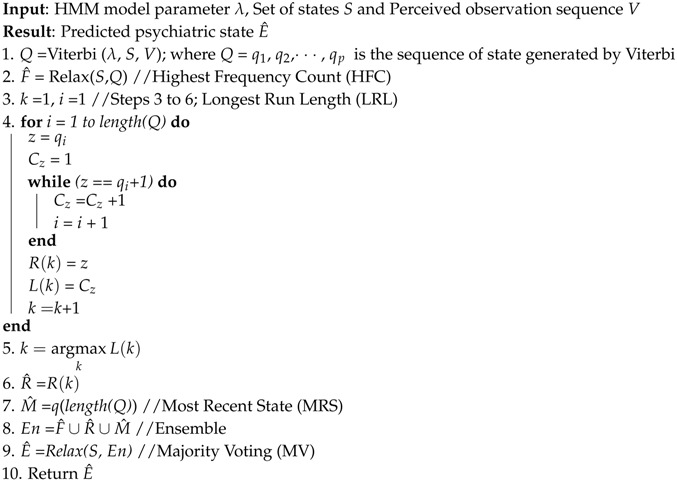


However, it is not realistic to return each time slot’s (e.g., every second) determined psychiatric state to the concerned consumers (e.g., psychiatrist). Therefore, the overall prediction of *T* time duration was required. Hence, from the Viterbi generated state sequence *Q*:

(a) The run lengths of each of the individual state were counted, and the state having the LRL was considered as the candidate state, as presented in Steps 3 to 6 of Algorithm 1 and in Row 2 of Table 1.
(b) The frequencies of each of the individual states in *Q* were counted, and the state having the HFC was considered as the candidate state as presented in Step 2 of Algorithm 1, Algorithm 2 and in Row 3 of Table 1.
(c) The state corresponding to the most recent time slot (i.e., $t_{T}$) was the MRS and was also considered as the candidate state as presented in Step 7 of Algorithm 1 and in Row 4 of Table 1.

**Algorithm 2:** The *Relax(S, Q)* procedure. 
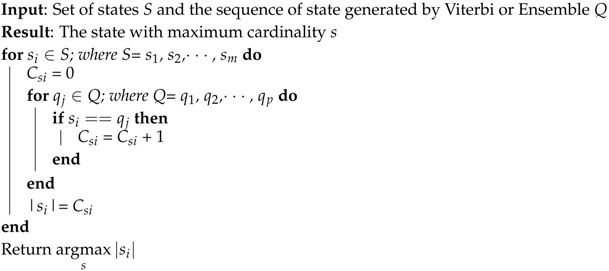


Afterwards, the ensemble of candidate states of HFC, LRL and MRS was prepared as presented in Step 8 of Algorithm 1 and in Row 5 of Table 1. Finally, the Majority Voting (MV) technique was applied in the ensemble of three candidate states and returned the candidate state with the maximum cardinality as the predicted psychiatric state (in Step 9 of Algorithm 1 and in Row 6 of Table 1).

## 4. Prototype Implementation and Performance Evaluation

This section broadly discusses the prototype implementation of the proposed AAL framework. Subsequently, it has also studied the performance of the proposed emergency psychiatric state prediction method.

### 4.1. Prototype Implementation

**Figure 11:**
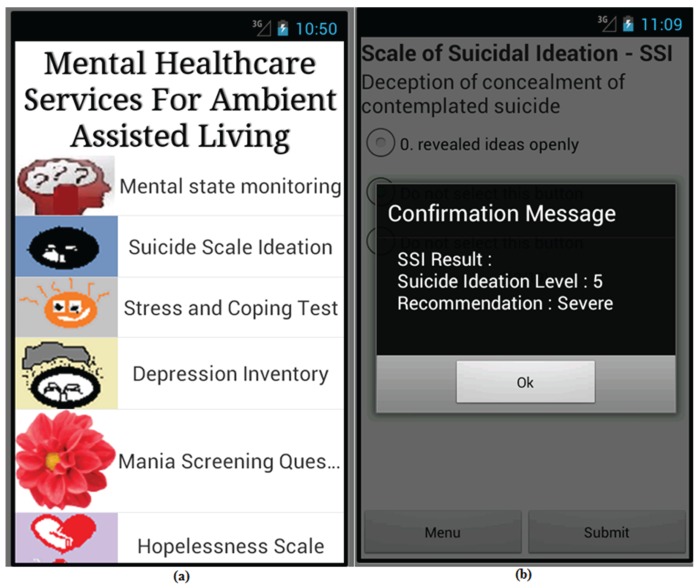
The prototype implementation of AAL framework: (**a**) Enabled mental healthcare services through the AAL framework; and (**b**) Evaluation result of benchmark Scale of Suicide Ideation (SSI) [30].

**Figure 12:**
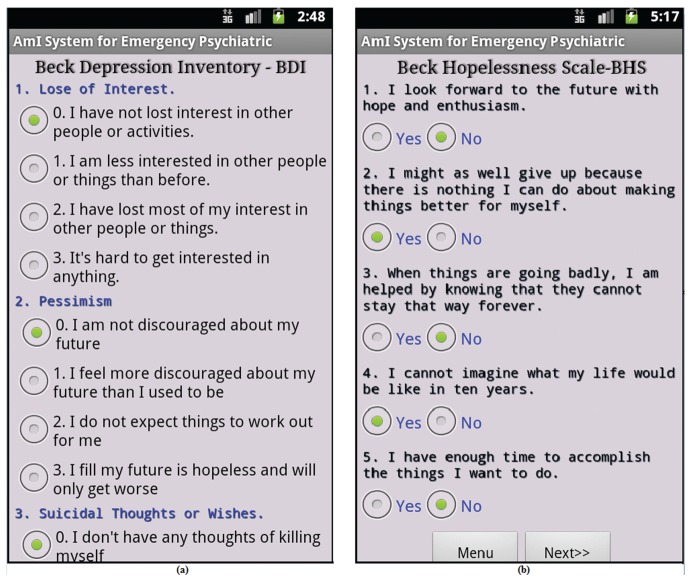
The benchmark questionnaire prototype: (**a**) Beck Depression Inventory (BDI) [10]; and (**b**) Beck Hopelessness Scale (BHS) [15].

To enable mental healthcare service in an AAL framework, four biosensors (EDA, ECG, EMG and BVP) were used to form a body sensor network, as shown in Figure 2, where a smartphone is used as the sink node. The biosensor nodes can support MAC and PHY layer communication functionalities of IEEE 802.15.4 [51]. Patients were also identified according to the sink nodes MAC and their social security numbers during the registration process for mental healthcare service. In addition, the sensor observations and patients’ psychiatric screening scores were also collected through the web-based benchmarked psychiatric questionnaire. The smartphone-based questionnaires of the Beck Depression Inventory (BDI-II) [10], Scale for Suicide Ideation (SSI) [30], Beck Hopelessness Scale (BHS) [15], Stress and Coping Self-Test (SCST) [45], Baratt’s Impulsiveness Scale (BIS) [28], Buss-Perry Aggression Questionnaire (BPAQ) [27], Buss-Durkee Hostility Inventory (BDHI) [29], Patient Health Questionnaire (PHQ-9) [20], Generalized Anxiety Disorder (GAD-7) [52], etc., were developed to collect subjective and objective psychiatric ratings of a patient as presented in Figure 11 and Figure 12.

The scores of these rating scales also indicate significant ailments related to an emergency psychiatric mental state. Patients are required to answer the test questions weekly, or bi-weekly, or monthly, or once in a lifetime, according to the guidelines of the benchmarked questionnaire. The sink node of the biosensor network communicates with the gateway node using a standard SOAP-HTTP binding [53].

**Figure 13:**
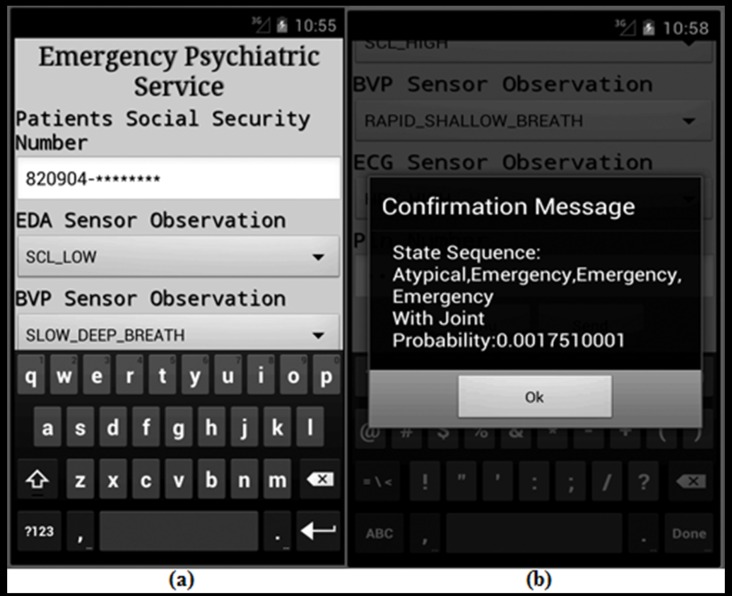
The AAL framework sends patient’s: (**a**) Biosensor observations; and (**b**) Returns an emergency psychiatric state sequence to the smart phone of a concerned psychiatrist.

**Figure 14:**
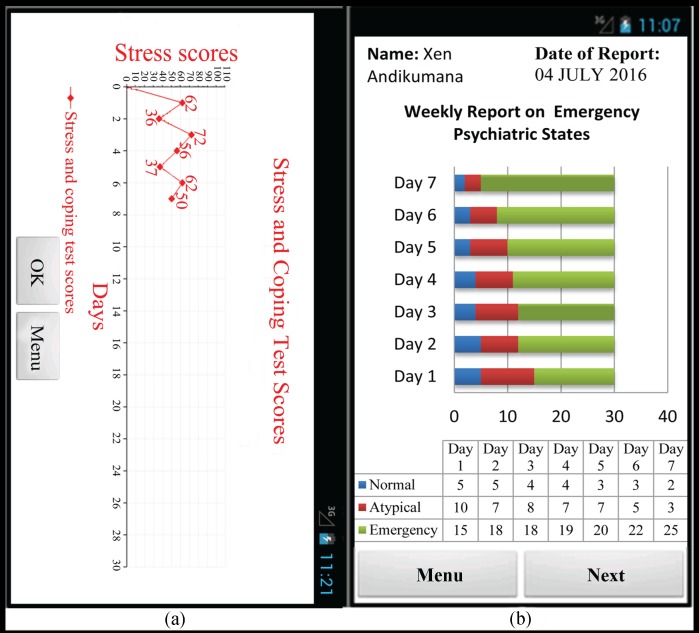
Mental health report generated through the WoO-based prototype AAL framework: (**a**) Monthly report on stress level; and (**b**) Weekly report on the prognosis of emergency psychiatric states.

The gateway modules are developed using GlassFish 4.1 [54] and servlet technology [55]. The object virtualization unit is developed by configuring a XenServer 6.5 [56] as the hypervisor in the AAL service platform. The semantic ontology for mental healthcare is developed in Protégé [57], and necessary object values are extracted from the developed ontology using SPARQL [58]. The inference engine of emergency psychiatric mental state prediction is developed using GlassFish 4.1 server technology. In case of a predicted psychiatric emergency, a mental healthcare service orchestrator proactively dispatches messages to a concerned authority as presented in Figure 13. The on-demand request from the legitimate authority is also honored by the service orchestrator, e.g., patients can get weekly reports of their stress level, and the concerned caregiver can submit a query regarding the prognosis of emergency psychiatric states, as shown in Figure 14.

### 4.2. Performance Evaluation

**Figure 15:**
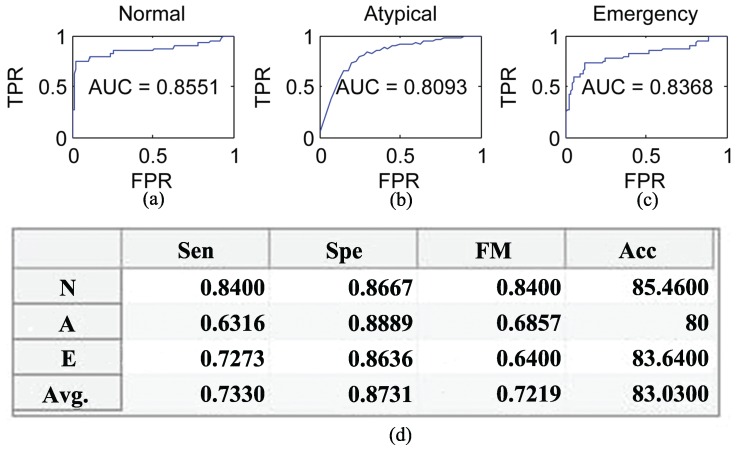
Area under the ROC curve of: (**a**) Normal, (**b**) Atypical and (**c**) Emergency psychiatric states; (**d**) The grid of sensitivity, specificity, F-measure and accuracy of different psychiatric states based on the testing dataset [36].

The collected dataset of 55 subjects was used to evaluate the performance of the proposed emergency psychiatric state prediction method. The psychiatric state prediction Accuracy (Acc), Sensitivity (Sen), Specificity (Spe), F-Measure (FM) and Area Under the ROC Curve (AUC) [59] of different psychiatric states are shown in Figure 15. However, the true positive rate against the false positive rate of the proposed mental state prediction model is compared with Mann, et al. [14] questioner-based traditional psychiatry model.

**Figure 16:**
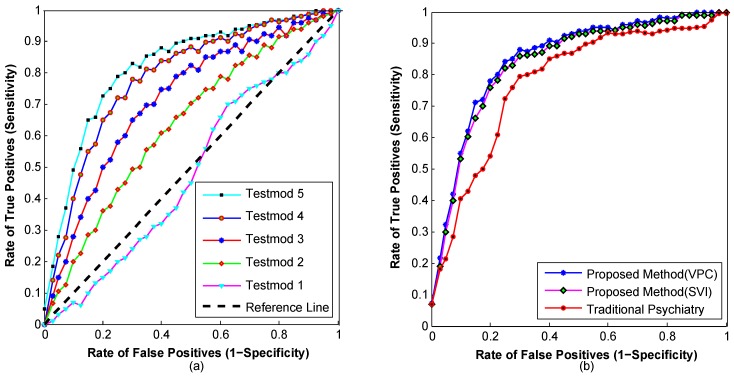
(**a**) Receiver Operating Characteristic (ROC) curves for presenting the true positive rate against the false positive rate of different test settings presented in Table 2; (**b**) ROC curve of psychiatric emergency state prediction using the proposed emergency psychiatry model (VPC and SVI training based) and the questioner-based traditional psychiatry model.

Table 2 presents the test settings to measure the performance of the proposed emergency psychiatric state prediction method as depicted in Figure 16. The measured sensitivity and specificity of each test case is also presented in Table 2.

Figure 16a depicts that using all of the sensors’ observations along with patients’ historical information from the semantic ontology repository, the prediction model of emergency psychiatric mental states has induced the highest sensitivity and specificity. The receiver operating characteristic curves as shown in Figure 16a demonstrate that just the family, demographic and medical histories are not sufficient for emergency psychiatric state prediction. The area under the ROC curve of Test Case 5 is 0.8368, which means that the use of biosensor observations with patients’ history enhances prediction accuracy.

The performance of the proposed emergency psychiatric state prediction model is also analyzed for VPC and the scalable SVI training algorithm. As shown in Figure 16b, the SVI-trained algorithm shows slightly lower ROC than the VPC-trained approach. This is because of the subsampling and mini-batch sizes of SVI-based HMM training. Here, the VPC uses the whole dataset in a single pass to learn HMM parameters, whereas the subsampling batch size is defined as 11 with a learning rate of 0.9 for SVI-based HMM training. Figure 16b also clearly shows the higher accuracy in psychiatric state prediction of the proposed method compared with the existing questioner-based clinical model. The continuous assessment is the key to such accuracy gain over the questioner-based methods.

The Type II or False Negative error Rate (FNR) is also studied with Type I or False Positive error Rate (FPR), as shown in Table 3. The TP, FP, TN and FN represent the number of True Positive, False Positive, True Negative and False Negative predictions, respectively, in the test settings [59]. The greater (≫1) Diagnostic Odds Ratio (DOR) and the positive 95 percent confidence interval of DOR indicate the proposed method’s higher discrimination capability among the psychiatric states.

Though the proposed model failed to address 100 percent of emergency cases, it reported the maximum portion (83%) of emergency psychiatric cases. However, modeling the psychiatric state through the hidden Conditional Random Field (CRF) [60] to represent the complex relations of considered features and the application of a Stepwise Linear Discriminant Analysis (SWLDA) [60] in PC features may enhance prediction accuracy.

## 5. Conclusions

A web of objects-based ambient assisted living framework is presented in this paper. In addition to the framework, a prediction method for emergency psychiatric state is also proposed. The psychophysiological and psychometric observations of inhabitants are collected through tiny biosensors and psychiatric screening scales and then objectified and virtualized to create intelligent service for ambient assisted living. The virtualized objects are shared for and participate in service composition and orchestration. The relationship among these objects is represented by semantic ontology. Therefore, the semantic ontology supplies the necessary interrelated objects to enable mental healthcare service and psychiatric emergency services. Although the average accuracy of psychiatric state prediction was 83.03%, the novel AAL framework and emergency psychiatric mental state prediction model can be used as the complement for the treatment of psychiatric patients in home environments.

Besides the proposed psychiatric state prediction approach, social network content-based sentiment analysis and abnormal behavior mining from the patient’s daily lifelog can be the complementary measures to provide a safety net for the future deployment of the proposed system. Furthermore, the ontology model presented in Section 3 and Figure 5 provides a platform for effective collaboration among different entities of safety nets (e.g., ‘psychiatrist’, ‘law enforcement agencies’ and ‘social worker associations’) to facilitate emergency care.

## Figures and Tables

**Table 1:** Illustration of the algorithm with an example.

Processing Steps	$t_{1}$	$t_{2}$	$t_{3}$	$t_{4}$	$t_{5}$	$t_{6}$	$t_{7}$	$t_{8}$	$t_{9}$	$t_{10}$	Candidate Results
Viterbi	N	N	A	E	E	E	A	E	E	A	-
LRL	N:2		A:1	E:3			A:1	E:2		A:1	E
HFC	N:2			A:3			E:5				E
MRS										A	A
Ensemble								E	E	A	-
MV								E:2		A:1	E

**Table 2:** Different test settings with performance evaluation.

Test Cases	Test Settings	Sensitivity	Specificity
Test Case 1	Without sensor observations (Only using patients’ medical, demographic and family histories)	0.4286	0.5177
Test Case 2	EDA sensor observations with patients’ history	0.6167	0.6493
Test Case 3	ECG and EDA sensors observation with patients’ history	0.6332	0.7281
Test Case 4	BVP, ECG and EDA sensors observation with patients’	0.6871	0.8014
Test Case 5	EMG, BVP, ECG and EDA sensors observation with patients’ history	0.7273	0.8636

**Table 3:** Type I and Type II error rate, DOR and CI of the predicted psychiatric states.

Psychiatric State	TP	FP	TN	FN	FPR = FP/(FP + TN)	FNR = FN/(FN + TP)	DOR = (TP × TN)/(FP × FN)	Confidence Interval (CI)
N	21	4	26	4	0.1333	0.16	34.125	(3.6, 6.7)
A	12	4	32	7	0.111	0.3684	13.714	(2.4, 5.2)
E	8	6	38	3	0.1364	0.2727	16.889	(2.5, 5.7)
				Average =	0.1269	0.2670		

## Abbreviations

A: Atypical
AAL: Ambient Assisted Living
Acc: Accuracy
AUC: Area Under the ROC Curve
BDI: Beck Depression Inventory
BVP: Blood Volume Pulse
BDHI: Buss–Durkee Hostility Inventory
BHS: Beck Hopelessness Scale
BIS: Baratt’s Impulsiveness Scale
BPAQ: Buss–Perry Aggression Questionnaire
BDHI: Buss–Durkee Hostility Inventory
CI: Confidence Interval
CRF: Conditional Random Field
DOR: Diagnostic Odds Ratio
DSM: Diagnostic and Statistical Manual of Mental Disorders
E: Emergency
EDA: Electro-Dermal Activity
ECG: Electrocardiogram
EMG: Electromyography
FNR: False Negative Error Rate
FPR: False Positive Error Rate
FM: F-measure
FP: False Positive
FN: False Negative
FH: Family History
GDA: Generalized Discriminant Analysis
GAD: Generalized Anxiety Disorder
HMM: Hidden Markov Model
HRSD: Hamilton Rating Scale for Depression
HFC: Highest Frequency Count
LS: Light Sensor
LRL: Longest Run Length
MRS: Most Recent Candidate State
mRMR: minimal-Redundancy-Maximal-Relevance
MV: Majority Voting
N: Normal
PH: Patients’ History
PHQ: Patient Health Questionnaire
PCA: Principal Component Analysis
ROC: Receiver Operating Characteristic
SOA: Service Oriented Architecture
SM: Smart Meter
SC: Smart Camera
SSI: Scale of Suicide Ideation
SCST: Stress and Coping Self-Test
SVI: Stochastic Variational Inference
SWLDA: Stepwise Linear Discriminant Analysis
Sen: Sensitivity
Spe: Specificity
TP: True Positive
TN: True Negative
TS: Temperature Sensor
VPC: Viterbi Path Count
WoO: Web of Objects
